# Palmitate Compromises C6 Astrocytic Cell Viability and Mitochondrial Function

**DOI:** 10.3390/metabo14030161

**Published:** 2024-03-12

**Authors:** Luisa O. Schmitt, Antonella Blanco, Sheila V. Lima, Gianni Mancini, Natalia F. Mendes, Alexandra Latini, Joana M. Gaspar

**Affiliations:** 1 Laboratory of Neuroimmune-Metabolism, Federal University of Santa Catarina, Florianópolis 88037-000, Brazil; 2 Graduate Program in Biochemistry, Federal University of Santa Catarina, Florianópolis 88037-000, Brazil; 3 Laboratory of Bioenergetics and Oxidative Stress (LABOX), Department of Biochemistry, School of Biological Sciences, Federal University of Santa Catarina, Florianópolis 88037-000, Brazil; 4 Centro de Investigaciones en Bioquímica Clínica e Inmunología (CIBICI-CONICET), Departamento de Bioquímica Clínica, Facultad de Ciencias Químicas, Universidad Nacional de Córdoba, Córdoba 5000, Argentina; 5 School of Medical Sciences, Department of Translational Medicine (Section of Pharmacology), University of Campinas, Campinas 13083-887, Brazil

**Keywords:** obesity, inflammation, mitochondria, saturated fatty acids, astrocytes

## Abstract

Consumption of high-fat diets (HFD) is associated with brain alterations, including changes in feeding behavior, cognitive decline, and dementia. Astrocytes play a role in HFD-induced neuroinflammation and brain dysfunction; however, this process is not entirely understood. We hypothesized that exposure to saturated fatty acids can compromise astrocyte viability and mitochondrial function. The C6 (astrocytes) cell line was treated with palmitate or stearate (200 µM and 400 µM) for 6 h. Cell viability, morphology, inflammatory markers, and oxidative stress were evaluated. To assess mitochondrial function, various parameters were measured (membrane potential, mass, respiration, and complex activities). We observed that 6 h of treatment with 400 µM palmitate decreased cell viability, and treatment with 200 µM palmitate changed the astrocyte morphology. Palmitate increased inflammatory markers (TNF-α and IL6) but did not induce oxidative stress. Palmitate significantly decreased the mitochondrial membrane potential and mitochondrial mass. Complex I activity also decreased in palmitate-treated cells; however, no changes were observed in mitochondrial respiration. In conclusion, palmitate, a saturated fatty acid, induces inflammation and impairs mitochondrial function, leading to reduced astrocytic cell viability and changes in cellular morphology. Our study provides valuable insights into the potential mechanisms underlying the relationship between saturated fatty acids, astrocytes, and mitochondrial function in obesity-related brain dysfunction.

## 1. Introduction

Lifestyle changes have occurred in recent decades, marked by an increased consumption of high-fat and high-sugar diets, coupled with sedentary behavior, leading to the development of obesity. As a result, the global prevalence of obesity has risen, with approximately 1.9 billion adults classified as overweight and 650 million as obese, according to the World Health Organization (WHO) [1].

Obesity is defined as the abnormal or excessive accumulation of fat, stemming from the dysregulation of whole-body energy homeostasis mechanisms [2]. Obesity and the consumption of high-fat diets are risk factors for metabolic and neurodegenerative diseases (for a review, see [3]). A high body mass index and chronic consumption of a high-fat diet are associated with detrimental neurochemical, structural, and behavioral alterations in the brain. These alterations are linked to conditions such as depression, cognitive decline, and dementia [4,5,6,7]. However, the pathophysiological and molecular mechanisms underlying the effects of obesity and high-fat diets on brain function have not been fully elucidated.

Mitochondria is the organelle responsible for maintaining energy homeostasis, mainly through oxidative phosphorylation, responsible for generating Adenosine triphosphate (ATP). Mitochondrial dysfunction plays a role in the pathophysiology of obesity, contributing to the dysregulation of energy homeostasis and the onset of chronic inflammation [8,9,10]. Obesity and associated chronic metabolic diseases exhibit lipotoxicity, a condition characterized by elevated levels of circulating saturated free fatty acids (FFAs). These FFAs can cross the blood–brain barrier, reaching the brain parenchyma and inducing neurotoxicity and neuroinflammation [11,12]. Animal models of obesity and obese patients’ brains show an increase in saturated FFAs, such as the proinflammatory palmitic acid [12].

Astrocytes, the most abundant cells in the central nervous system, play essential roles in supporting neuronal function, transporting substances across the blood–brain barrier, storing energy, regulating neurotransmission, and participating in the immune regulation of the brain. Astrocytes can maintain brain homeostasis by sensing nutrients, hormones, and other metabolites in response to metabolic alterations. They become reactive in response to injurious stimuli, such as stroke, hypoxia, infection, neurodegenerative diseases, and excitotoxicity [13,14]. Astrocytes play a role in high-fat diets (HFD)-induced neuroinflammation, although this process is not entirely understood. Palmitate induces various metabolic and molecular changes in astrocytes, contributing to pathological conditions related to neurodegenerative diseases.

Chronic exposure to high-fat diets leads to an increase in the glial fibrillary acid protein (GFAP) protein in the astrocytes of rodents and humans, indicating the presence of astrogliosis even before an increase in body weight [15,16]. Palmitate induces several metabolic and molecular changes, contributing to pathological conditions related to neurodegenerative diseases. The treatment of astrocyte cells with palmitate increases glutathione, interferes with the hypothalamic synaptic network [17], and impairs autophagy [18]. Palmitic acid significantly decreases glucose transporter (GLUT1) levels, down-regulating glucose uptake and lactate release by astroglia [14]. Palmitic acid induces apoptosis by increasing oxidative stress in neurons and astrocytes [19]. However, the effect of saturated fatty acids on mitochondrial function in astrocytes is not yet fully understood.

In this work, our main aim was to study the effect of saturated fatty acids, specifically palmitate (C16:0) and stearate (C18:0), on astrocytic cell viability, inflammation, and mitochondrial function. We demonstrated that palmitate, but not stearate, decreases cell viability, induces inflammation, and reduces mitochondrial membrane potential, along with changes in mitochondrial complexes’ activity. Alterations in astrocyte physiology and metabolism may pose a risk factor for neuronal dysfunction in the context of chronic obesity and high-fat diet consumption.

## 2. Materials and Methods

### 2.1. Materials

Reagents were acquired from Sigma, St. Louis, MO, USA, unless otherwise specified in the text. The reagents for cell culture were obtained from Gibco^®^ (Gaithersburg, MD, USA).

### 2.2. Cell Culture

We utilized the astroglioma cell line C6 obtained from the American Type Culture Collection (Rockville, MD, USA). This cell line exhibits astrocytic characteristics, with 99% of cells expressing GFAP. Cells were cultured in low glucose Dulbecco’s Modified Eagle Medium (DMEM), supplemented with 10% (*v*/*v*) fetal bovine serum (FBS) and 1% (*v*/*v*) antibiotic–antimycotic solution. The cell culture was maintained in a humidified incubator with 5% CO_2_/95% air at 37 °C. Upon reaching 85% confluence, cells were trypsinized with 0.05% trypsin–EDTA (ethylenediaminetetraacetic acid) and then plated in 12-well or 96-well plates (75,000 cells/mL) for 24 h. After 24 h in culture, cells were incubated with 200 µM and 400 µM palmitic acid or 200 µM and 400 µM stearic acid for 6 h. Control cells were incubated with 2.5% bovine serum albumin (BSA).

### 2.3. Fatty Acid Conjugation with BSA

Fatty acids were conjugated with BSA (free of fatty acids), freshly prepared before experiments, following the guidelines and recommendations of Alsabeeh and colleagues [20]. Briefly, a solution of 10 mM stearate and 10 mM palmitate was prepared in water and heated to 65 °C until the solution became completely clear. Simultaneously, a 10% (*m*/*v*) BSA solution was prepared in water at 37 °C. Fatty acid/BSA conjugation was carried out by incubating fatty acids with albumin at a 3:1 ratio (following the physiological ratio) at 37 °C for 60 to 90 min with occasional shaking.

### 2.4. Assessment of Cell Viability

Cell viability was assessed using the 3-(4,5-dimethylthiazol-2-yl)-2,5-diphenyltetrazolium (MTT) assay. Cells were washed with 1× phosphate-buffered saline (PBS) solution (137 mM NaCl, 2.7 mM KCl, 0.5 mM MgCl_2_, 1.0 mM CaCl_2_, 10 mM Na_2_HPO_4_, 2 mM KH_2_PO_4_; pH 7.4) and then incubated with MTT (0.5 mg/mL) solubilized in PBS for 1 h at 37 °C in the incubation chamber. After incubation, the medium was removed, and the precipitated dye was dissolved in dimethylsulfoxide (DMSO) and quantified colorimetrically (absorbance at 540 nm) using a microplate reader (Infinite M200 TECAN, Life Sciences, Männedorf, Switzerland, equipment from the Laboratório Multiusuário de Estudos em Biologia at the Universidade Federal de Santa Catarina-LAMEB/UFSC). All experiments were carried out in triplicate, and cell viability was expressed as a fraction of the control. Reagents were acquired from Sigma, St. Louis, MO, USA.

### 2.5. Assessment of Cellular Morphology

Cell morphology was assessed by immunocytochemistry for an astrocyte cell marker, GFAP. Briefly, cells were washed twice with warm PBS and fixed with 4% paraformaldehyde containing 4% sucrose for 10 min at room temperature. After rinsing twice in PBS, cells were permeabilized with 1% Triton X-100 in PBS* (PBS supplemented with 0.03% BSA plus 0.02% sodium azide) for 10 min. Following a 30 min block with 10% horse serum in PBS*, cells were incubated with the primary antibody (GFAP, 1:500, Abcam #ab7260) for 2 h at room temperature. Subsequently, cells were rinsed three times with PBS*, followed by incubation for 1 h at room temperature in the dark with an Alexa Fluor 488-conjugated secondary antibody (anti-rabbit IgG, 1:500). The preparations were visualized using fluorescence microscopy (IX83 Inverted Microscope, Olympus, Life Science Solutions, equipment from LAMEB/UFSC).

### 2.6. RNA Extraction and Quantitative Real-Time PCR (qRT-PCR)

Total RNA was extracted from cellular extracts using TRIzol reagent (Sigma) following the manufacturer’s recommendations. The cDNA synthesis was performed with 2.0 μg of total RNA according to the manufacturer’s instructions (High Capacity cDNA Reverse Transcription Kit, Life Technologies, Carlsbad, CA, USA). Each PCR reaction included 25 ng of reverse-transcribed RNA, 0.25 μL of each specific primer, Taqman Probe Mix (PCR Biosystems, London, UK), and RNAse-free water, resulting in a final volume of 8 μL. Real-time PCR analysis of gene expression was conducted using a QuantStudio 6 Flex Real-Time PCR System (Applied Biosystems, Waltham, MA, USA). Primers were sourced from Applied Biosystems or Integrated DNA Technologies. For RT-PCR calculation, we employed delta CT, and relative gene expression was normalized to that of glyceraldehyde-3-phosphate dehydrogenase (GAPDH) in all samples. Gene bank numbers for all primers are listed in Table 1.

### 2.7. Mitochondrial Membrane Potential

Changes in mitochondrial membrane potential were determined using the JC-1 (Thermo Fisher Scientific, Waltham, MA, USA) probe and the fluorescent probe MitoTracker Deep Red (Thermo Fisher Scientific, Waltham, MA, USA). JC-1 is a cationic fluorescence probe which selectively accumulates either within the mitochondrial matrix as a green (530 nm) fluorescent monomer at depolarized membrane potentials or as J-aggregates with orange–red (590 nm) fluorescence at hyperpolarized membrane potentials. Briefly, C6 cells were seeded for 24 h and treated for 6 h with saturated fatty acids. Afterward, the cells were washed with pre-warmed 1x PBS and incubated with pre-warmed 5 µg/mL JC-1 for 30 min at 37 °C. Cells were then washed with 1x PBS, and fluorescence was read by spectrophotometry with excitation at 485 nm and 535 nm and emission at 530 nm and 590 nm, respectively, using a microplate reader (Infinite M200 TECAN, Life Sciences, Männedorf, Switzerland, equipment from LAMEB/UFSC). The loss of mitochondrial membrane potential was reflected by the ratio of aggregates (red fluorescence) to monomers (green fluorescence).

For the fluorescent probe MitoTracker Deep Red, C6 cells were seeded for 24 h and treated for 6 h with saturated fatty acids. Subsequently, the cells were washed with pre-warmed 1x PBS and incubated with pre-warmed MitoTracker staining solution (diluted in DMEM without serum to a final concentration of 300 nM) for 15 min at room temperature. All subsequent steps were performed in the dark. Cells were washed in PBS, fixed in 4% paraformaldehyde with 4% sucrose for 5 min, washed in PBS, and nuclei were stained with 5 µg/mL 4′,6-diamidino-2-phenylindole (DAPI) for 5 min. Mitochondria were visualized at 620/700 nm, and nuclei were visualized at 350/460 nm using fluorescence microscopy (IX83 Inverted Microscope, Olympus, Life Science Solutions, equipment from LAMEB/UFSC). All experiments were conducted in triplicate, acquiring 6 images for each well. Total fluorescence was quantified using ImageJ^®^ software (National Health Institute, USA) for all images. Quantification was performed using a ratio of the intensity of MitoTracker Deep Red per the number of nuclei, and data were expressed as a percentage of the control.

### 2.8. Measurement of Mitochondrial Respiration

Oxygen consumption rates were polarographically measured using a high-resolution respirometry apparatus (Oxygraph-O2K, OROBOROS Instruments, Innsbruck, Austria). C6 cells adhered to the plates during growth. After a 6 h treatment with palmitate/vehicle, we trypsinized the cells and resuspended them in serum-free DMEM. After that cell count, 500,000 cells/mL were diluted in respiration and added to the Oroboros chamber. Air calibration was carried out before every experiment and in accordance with equipment instructions. The measurements were conducted in medium DMEM low-glucose without FBS at 37 °C. In brief, 500,000 cells/mL, previously treated with 200 µM palmitate for 6 h or with a vehicle, were added in the recording chamber for stabilization. Baseline cellular oxygen consumption rates (OCR) is measured, from which basal respiration can be derived by subtracting non-mitochondrial respiration. Measurements were carried out with continuous stirring. After signal stabilization, the experimental protocol involved the sequential addition of 1.2 μM oligomycin (complex V inhibitor), titration with 2.5–4 μM carbonyl cyanide-4-(trifluoromethoxy)-phenylhydrazone (FCCP) until reaching the maximal respiratory capacity, 0.5 μM rotenone, and 2.4 μM antimycin A. Oligomycin was added and the resulting OCR was used to derive ATP-linked respiration and proton leak respiration. Next FCCP, a protonophore, was added to collapse the inner membrane gradient, allowing the electron transport chain to function at its maximal rate, and the maximal respiratory capacity was derived by subtracting non- mitochondrial respiration from the FCCP rate. Lastly, antimycin A and rotenone (inhibitors of complex III and I, respectively) were added to shut down the electron transport chain function, revealing the non-mitochondrial respiration. Mitochondrial reserve capacity is calculated by subtracting basal respiration from the maximal respiratory capacity. DatLab software 7.0 (Oroboros Instruments, Innsbruck, Austria) was utilized for data acquisition and analysis.

### 2.9. Measurement of Mitochondrial Respiratory Complex Activity

For the determination of the respiratory chain complex I activity, the method from Cassina and Radi was employed with modifications [21]. The basis of this method is to observe the decrease in absorbance at 420 nm as a result of the reduction of ferricyanide K_3_[Fe(CN)_6_] which is converted to ferrocyanide K_4_[Fe(CN)_6_], by Nicotinamide adenine dinucleotide (NADH) dehydrogenase from NADH. Samples (approximately 0.2–0.4 mg of proteins) were introduced to a reaction medium containing 100 mM sodium phosphate buffer (pH 7.4), 80 μM rotenone, and 500 μM FeCN. The reaction initiated after adding 0.35 mM NADH and was monitored for 5 min by spectrophotometry (Multireader Infinite M200 TECAN).

For the determination of respiratory chain complex II activity, the reduction of 2,6-dicloindophenol (DCIP) by succinate dehydrogenase at 600 nm was measured, following the method described by Fischer [22]. Samples were incubated for 20 min at 37 °C in a reaction medium containing 62.0 mM sodium phosphate buffer (pH 7.4), 9.2 mM sodium succinate, and 5.0 μM DCIP. After incubation, 2.5 mM sodium azide and 40.0 μM rotenone were added, and the reaction was initiated by adding 25 μM DCIP. It was monitored for 5 min at 37 °C by spectrophotometry (Multireader Infinite M200 TECAN). The results are expressed in nmol of DCIP reduced per mg of protein per minute, using the molar extinction coefficient of 20.5 mM^−1^ cm^−1^ for DCIP. Complex II activity was calculated as nmol/min/mg protein.

The respiratory chain complex IV activity was determined according to Rustin [23], calculated by the decrease in absorbance caused by reduced cytochrome c oxidation measured at 550 nm over 5 min by spectrophotometry (Multireader Infinite M200 TECAN). Samples (about 0.2–0.4 mg of proteins) were added to a reaction medium containing 200 mM Tris–HCl buffer (pH 7.4) plus the exact volume of reduced cytochrome c solution (1.3 mM) needed to have a final concentration of 30 μM in the medium. The results were expressed in cytochrome c nmol reduced per mg of protein per minute, using the molar extinction coefficient of 19 × 103 M^−1^ cm^−1^ for cytochrome c. Complex IV activity was calculated as nmol/min/mg protein.

### 2.10. ROS Generation

ROS generation was assessed using the molecular probe 2,7-dichlorodihydrofluorescein diacetate (DCFH-DA, Sigma-Aldrich, St. Louis, MO, USA). This probe diffuses through the cell membrane and is hydrolyzed by intracellular esterases to the non-fluorescent form, dichlorodihydrofluorescein (DCFH). DCFH reacts with intracellular ROS to form dichlorofluorescein (DCF), a green fluorescent dye, with the intensity of DCF fluorescence being proportional to the amount of ROS. C6 cells were incubated with DCFH-DA (200 μM) for 30 min at 37 °C, washed, and fluorescence was measured. All experiments were conducted in triplicate, using excitation and emission wavelengths of 480 and 525 nm, respectively, with a microplate reader (Infinite M200 TECAN, Life Sciences, Männedorf, Switzerland, equipment from LAMEB/UFSC). The results were expressed in concentration (µM). Reagents were acquired from Sigma, St. Louis, MO, USA.

### 2.11. TBARS—Thiobarbituric Acid Reactive Species Levels

The TBARS assay was used to quantify levels of substances reactive to thiobarbituric acid (TBA). Initially, 50 μL of the cell lysate was subject to 100 μL of trichloroacetic acid 10%, followed by centrifugation at 300× *g* for 10 min to precipitate the proteins. Then, the supernatant was transferred to a glass tube containing 150 μL of TBA 0.67% and boiled in a water bath for 1 h at 95 °C. To cool the samples, they received an ice bath for 5 min. Next, 400 μL of n-butanol was added to the samples in Eppendorf tubes, and the samples were centrifuged for 5 min at 5.000× *g.* Finally, 200 μL of the organic phase was transferred to a microplate, and the fluorescence was measured at a wavelength excitation of 515 nm and wavelength emission of 553 nm. Samples concentrations were calculated based on the TBARS levels using the linear standard curve of 1,1,3,3 tetraethoxypropane. Reagents were acquired from Sigma, St. Louis, MO, USA.

### 2.12. Statistical Analysis

Data are presented as means ± SEM. Statistical significance was assessed by an analysis of variance (ANOVA), followed by Dunnett’s post hoc test. Differences were considered significant for *p* < 0.05.

## 3. Results

### 3.1. Effect of Saturated Fatty Acids on Cellular Viability and Morphology in Astrocytes

**Figure 1:**
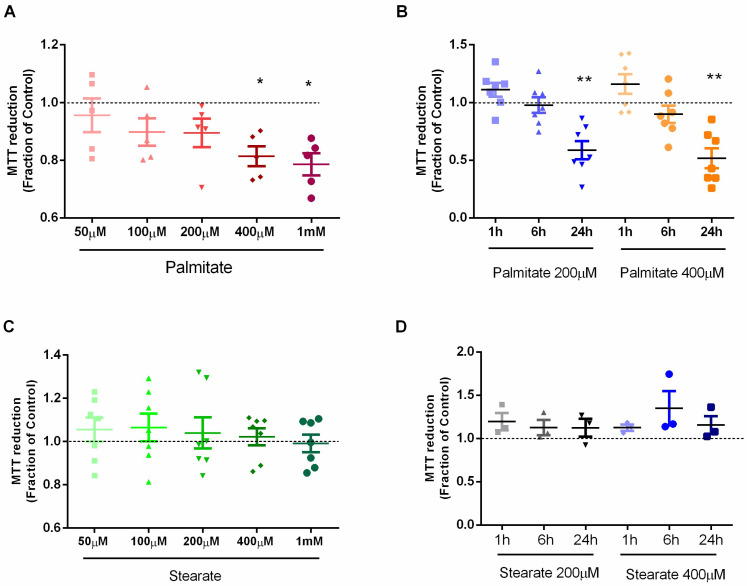
Effect of saturated fatty acids on astrocytic cell viability. Cell viability was evaluated by MTT assay after treatment with saturated fatty acids. (**A**) Viability of astrocytes after treatment with different concentrations of palmitate for 6 h; (**B**) Time curve of astrocyte viability after treatment with 200 µM and 400 µM palmitate; (**C**) Viability of astrocytes after treatment with different concentrations of stearate for 6 h; (**D**). Time curve of astrocyte viability after treatment with 200 µM and 400 µM stearate. *n* = 4–7; * *p* ≤ 0.05 and ** *p* ≤ 0.01, compared to the control.

Cell viability was assessed using the MTT assay. A dose–response curve showed that cells treated with 400 µM and 1 mM of palmitate had a significantly decreased astrocyte viability (Figure 1A). In contrast, cells treated with different concentrations of stearate did not show changes in cellular viability (Figure 1C).

As palmitate concentrations below 400 µM showed a low effect on viability, concentrations of 200 µM and 400 µM were chosen to evaluate the effect on cell viability over time. After 1 h and 6 h treatment, both concentrations did not affect cellular viability. However, after 24 h of treatment, there was a 50% reduction in cellular viability with both concentrations (Figure 1B). In contrast, stearate has no effects on cellular viability over 24 h of treatment (Figure 1D). Because the concentration of 200 µM palmitate and 200 µM stearate did not cause any effects on viability after 6 h of treatment, this concentration and this time were chosen for the treatments in the following experiments.

**Figure 2:**
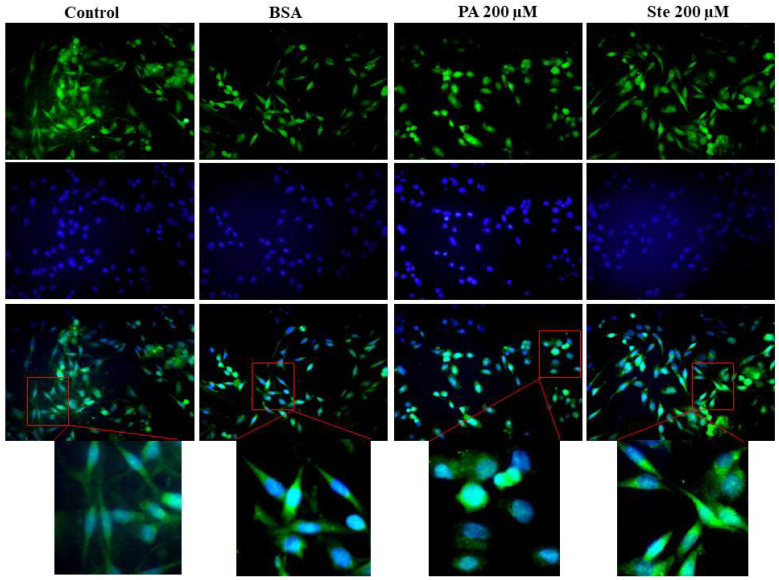
Effect of saturated fatty acids in the cell morphology of astrocytes. Cell morphology was evaluated by immunocytochemistry, using the GFAP marker (green) and DAPI as a nuclear marker (blue). The morphology of astrocytes after treatment with 200 µM palmitate (PA) or 200 µM stearate (Ste) for 6 h. Inserts represents a magnified crop of cells. Representative images of *n* = 3.

The effect of saturated fatty acids on the astrocytic cell morphology was analyzed by GFAP immunocytochemistry. It was observed that 200 µM palmitate for 6 h of treatment changes the morphology of astrocytes which changed from a stellate phenotype with fibroblastic characteristics to a rounded shape (Figure 2). These results suggest that palmitate treatment was responsible for the reactivation of astrocytes due to the change in cellular phenotype. But the stearate did not changed the astrocytes’ morphology.

### 3.2. Palmitate Increases Gene Expression of Pro-Inflammatory Cytokines in Astrocytes

**Figure 3:**
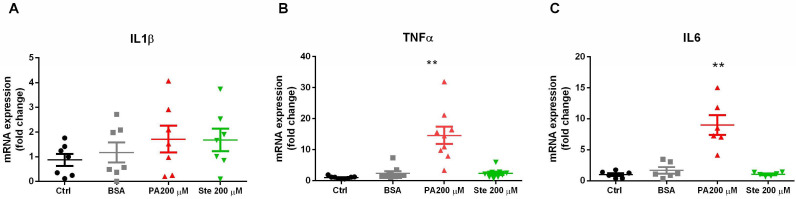
Effect of palmitate (PA) and stearate (Ste) treatment in the expression of pro-inflammatory cytokines. The C6 cells were exposed to 200 µM palmitate or 200 µM stearate for 6 h and then harvested for measurements of the transcript expression of pro-inflammatory cytokines by PCR. (**A**) Gene expression of IL-1β; (**B**) gene expression of TNF-α; and (**C**) gene expression of IL-6. The results were standardized by quantifying GAPDH gene expression. *n* = 6–9; ** *p* ≤ 0.01, compared to the control.

To evaluate the effect of saturated fatty acids on the induction of gliosis and inflammation, we measured the gene expression of pro-inflammatory cytokines by PCR. It was observed that treatment with 200 µM palmitate for 6 h significantly increased the gene expression of TNF-α and Interleukin 6 (IL-6) (Figure 3B,C) but did not change the expression of interleukin 1 β (IL-1β) (Figure 3A). However, the treatment with stearate did not change the gene expression of these pro-inflammatory cytokines. These results suggest that palmitate was able to increase the gene expression of pro-inflammatory cytokines, indicating that palmitate induces gliosis and has pro-inflammatory potential.

### 3.3. Palmitate Reduces Mitochondrial Membrane Potential in Astrocytes

**Figure 4:**
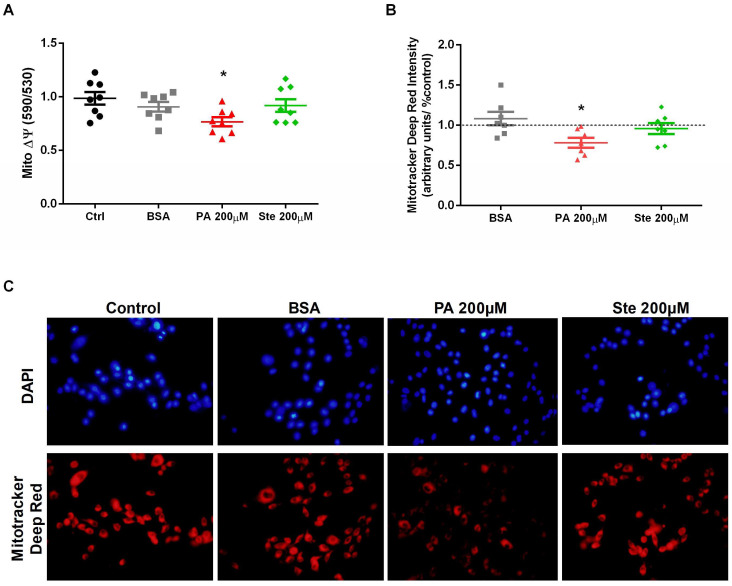
Effect of palmitate (PA) and stearate (Ste) treatment in mitochondria membrane potential (ψ). The C6 cells were exposed to 200 µM palmitate or 200 µM stearate for six hours and then mitochondrial membrane potential was measured. (**A**) Mitochondrial membrane potential measured by JC-1 dye at the emission absorbance (530 nm and 590 nm) after treatment with 200 µM palmitate (PA) or 200 µM stearate (Ste); JC-1 forms aggregate (in healthy mitochondria) with red fluorescence (590 nm). As the membrane potential decreases, JC-1 becomes a monomer, which shows green fluorescence (530 nm). The change in the ratio of red to green fluorescence is used as an indicator of mitochondrial condition. (**B**) Mitochondrial membrane potential measured by the fluorescent probe Mitotracker Deep Red (red) after treatments with saturated fatty acids. (**C**) Representative images of microscopy of Mitotracker Deep red (red) and DAPI (blue). *n* = 7–8; * *p* ≤ 0.05 compared to the control.

The mitochondrial membrane potential was evaluated by the fluorescent probes JC-1 and Mitotracker Deep Red. JC-1 forms aggregates (in healthy mitochondria) with red fluorescence (590 nm). As the membrane potential decreases, JC-1 becomes a monomer, which shows green fluorescence (530 nm). The change in the ratio of red to green fluorescence is used as an indicator of mitochondrial condition. Treatment with 200 µM palmitate significantly decreased the mitochondrial membrane potential evaluated by both the JC-1 probe (Figure 4A) and the Mitotracker Deep Red probe (Figure 4B,C). However, treatment with 200 µM stearate did not alter the mitochondrial membrane potential. These results indicate that palmitate was able to reduce the mitochondrial membrane potential, which is an essential parameter for maintaining the physiological function of the respiratory chain, being suggestive of mitochondrial dysfunction.

### 3.4. Palmitate Decreases the Expression of Mfn2 and Citrate Synthase (CS)

**Figure 5:**
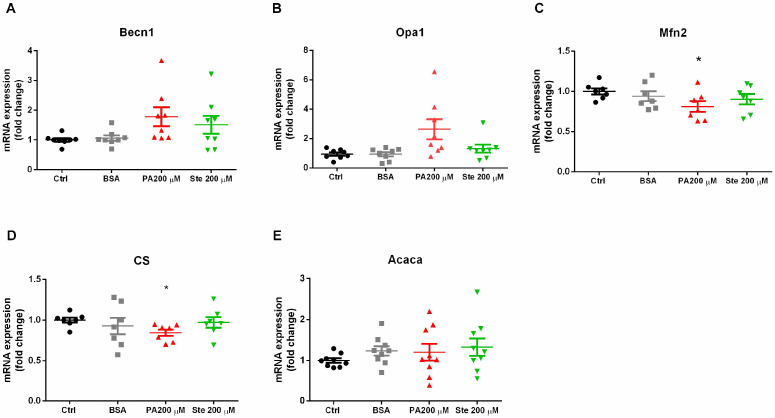
Gene expression of proteins involved in mitochondrial physiology. The C6 cells were exposed to 200 µM palmitate or 200 µM stearate for six hours and then harvested for measurements of transcript expression of mitochondrial proteins by PCR. (**A**) Gene expression of becn1 (beclin-1); (**B**) gene expression of Opa1; (**C**) gene expression of mfn2 (mitofusin-2); (**D**) gene expression of CS (citrate synthase); and (**E**) gene expression of Acaca (acetyl CoA carboxylase). The results were standardized by quantifying GAPDH gene expression. *n* = 6–9; * *p* ≤ 0.05, compared to the control.

To evaluate mitochondrial physiology, we measured the transcripts of some genes involved in mitochondrial physiological processes. We observed that treatment with 200 µM palmitate and 200 µM stearate did not alter the gene expression of becn1 (beclin, Figure 5A), a protein that is involved in macroautophagy, being an indicative that mitophagy is not altered by this concentration of saturated fatty acids for 6 h.

Mitochondrial fusion was assessed by gene expression of the Opa1 (located in the inner mitochondrial membrane) and mitofusin-2 (Mfn2, located in the outer mitochondrial membrane). Gene transcripts of Opa1 were not altered by treatments with 200 µM palmitate and 200 µM stearate (Figure 5B). However, the gene expression of Mfn2 significantly decreased after 6 h treatment with 200 µM palmitate (Figure 5C). This is suggestive that palmitate compromises adequate mitochondrial fusion. Stearate had no effects on either Opa1 or Mfn2.

Citrate synthase (CS) is a mitochondrial enzyme involved in the Krebs cycle and can be used as a marker of mitochondrial mass. Treatment with 200 µM palmitate significantly decreased the gene expression of CS. Stearate had no effects on citrate synthase (Figure 5D). These results suggest that palmitate can decrease the mitochondrial mass and can also cause metabolic dysregulation, as this enzyme is crucial for maintaining metabolism. Saturated fatty acids did not cause changes in the gene expression of the enzyme Acaca (acetyl-CoA carboxylase), indicating that the endogenous synthesis of fatty acids was not impaired (Figure 5E).

### 3.5. Palmitate Decreases the Activity of Mitochondrial Complex I

**Figure 6:**
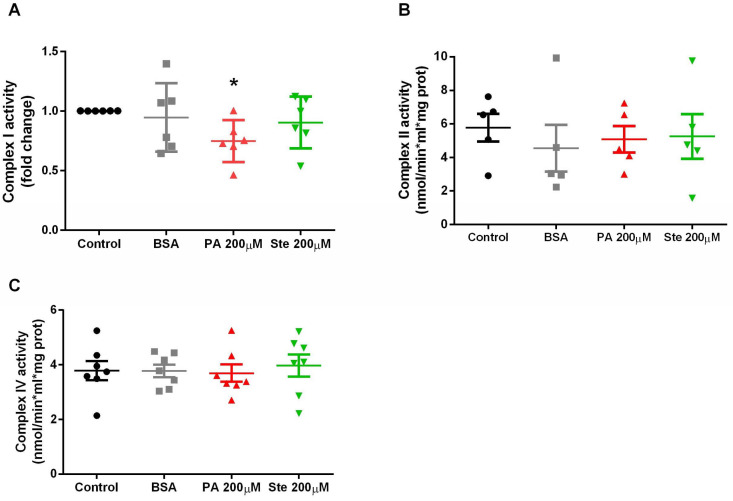
Enzymatic activity of complex I, complex II, and complex IV of the mitochondrial respiratory chain. (**A**) Mitochondrial complex I activity in astrocytes treated with palmitate (PA) and stearate (Ste); (**B**) Mitochondrial complex II activity in astrocytes treated with palmitate and stearate; (**C**) Mitochondrial complex IV activity in astrocytes treated with palmitate and stearate. *n* = 5–6; * *p* ≤ 0.05 compared to control.

The activities of the mitochondrial complex I, complex II, and complex IV were measured by enzymatic activity spectrometry. We observed that 6 h of 200 µM palmitate treatment significantly reduced the enzymatic activity of mitochondrial complex I (NADH dehydrogenase) (Figure 6A). However, no changes were observed for the enzymatic activity of complex II (Figure 6B) or complex IV (Figure 6C). This result suggests that the activity of the first enzyme involved in electron transport in the respiratory chain is impaired, which may favor the development of changes in mitochondrial membrane potential. Stearate treatment did not cause significant changes in the enzymatic activity of mitochondrial complexes.

### 3.6. Palmitate Does Not Change the Mitochondrial Respiration in Astrocytes

**Figure 7:**
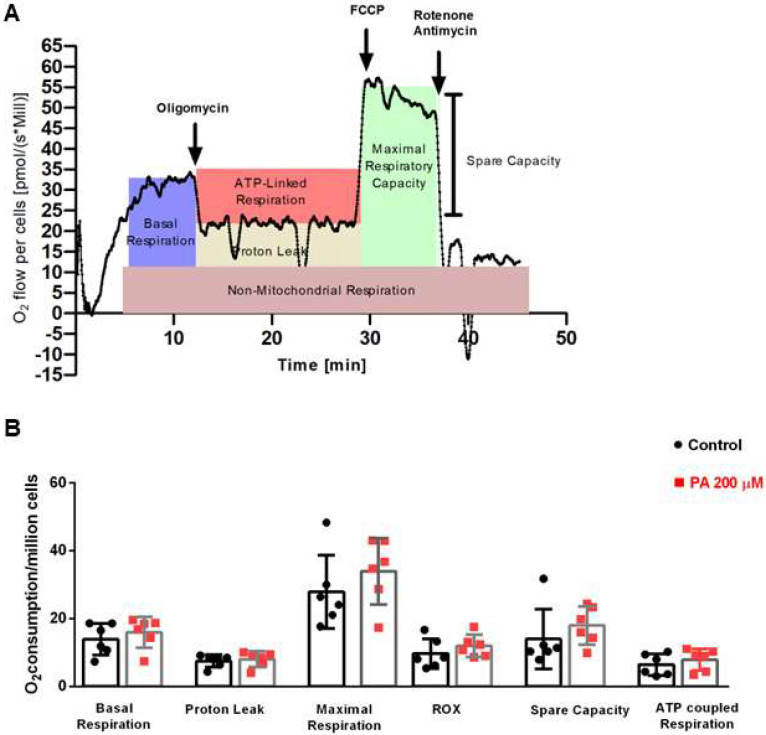
Mitochondrial oxygen consumption in astrocytes treated with palmitate. (**A**) Baseline cellular OCR was measured, from which basal respiration can be derived by subtracting non-mitochondrial respiration. Then, oligomycin (complex V inhibitor) was added and the resulting OCR was used to derive ATP-linked respiration and proton leak respiration. Next FCCP, a protonophore, was added to collapse the inner membrane gradient, allowing the electron transport chain to function at its maximal rate, and the maximal respiratory capacity was derived by subtracting non- mitochondrial respiration from the FCCP rate. Lastly, antimycin A and rotenone (inhibitors of complex III and I) were added to shut down the electron transport chain function, revealing the non-mitochondrial respiration. Mitochondrial reserve capacity is calculated by subtracting basal respiration from the maximal respiratory capacity. (**B**) Oxygen consumption per million cells in the parameters of basal respiration, proton leak, maximum respiration, extramitochondrial respiration (ROX), reserve capacity, and respiration coupled to ATP synthesis in astrocytes treated with palmitate (PA). *n* = 5.

Mitochondrial oxygen consumption was assessed by the Oroboros 2K high-resolution oxygraph. Treatment with 200 µM palmitate did not change oxygen consumption in basal respiration, state IV, maximal respiration, extramitochondrial oxygen consumption, mitochondrial reserve capacity, or ATP-coupled respiration, suggesting that mitochondrial respiration is not compromised (Figure 7).

### 3.7. Palmitate Does Not Induce Oxidative Stress in Astrocytes

**Figure 8:**
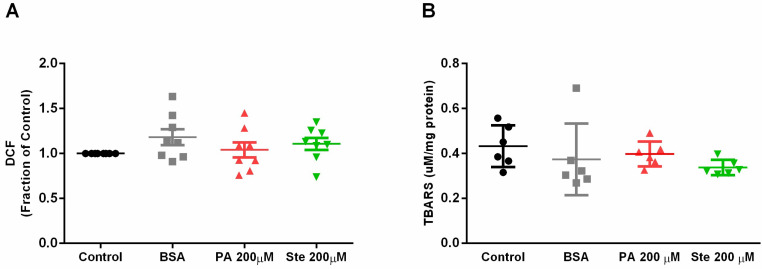
Assessment of reactive oxygen species and substances reactive to thiobarbituric acid. (**A**) DCF fluorescence levels as a percentage of the control in astrocytes treated with palmitate (PA) and stearate (Ste); (**B**) Levels of thiobarbituric-acid-reactive substances (TBARS) in astrocytes treated with palmitate (PA) and stearate (Ste). *n* = 6–7.

Oxidative stress was analyzed by the fluorescent probe DCF-DA and by a TBARS assay. Treatments with 200 µM palmitate and 200 µM stearate did not cause significant changes in dichlorofluorescein (DCF) fluorescence (Figure 8A) nor in the levels of substances reactive to thiobarbituric acid (Figure 8B), indicating that these saturated fatty acids do not alter the levels of reactive oxygen species or lipid peroxidation.

## 4. Discussion

The exact role of glial cells in the onset and progression of obesity-induced cognitive decline remains unclear. Considering the association between neuroinflammation, oxidative stress, and mitochondrial dysfunction, we hypothesized that saturated fatty acids induce astrocytic inflammation and mitochondrial dysfunction. In the present study, we used saturated fatty acids—palmitate, the most abundant saturated fatty acid in the diet, and stearate—to mimic a high-fat diet and obesity conditions. We demonstrated that the saturated fatty acid palmitate induces an inflammatory profile and reduces mitochondrial membrane potential and mitochondrial mass, affecting astrocytic viability and metabolism.

Palmitate and stearate are saturated fatty acids present in diets rich in ultra-processed foods usually called “cafeteria diets”. Cafeteria diets are hypercaloric and have low nutrients. Some studies have demonstrated that saturated fatty acids, such as palmitate and stearate, cross the blood–brain barrier (BBB) [24,25], estimating that approximately 5% of plasma palmitate crosses the BBB and reaches the brain unidirectionally in a single passage [25]. Palmitate and stearate are the saturated fatty acids most often found in the brains of mice treated with a high-fat Western diet, both in male and female mice [26]. However, central nervous system (CNS) cells have peculiarities in their ability to use substrates in oxidative metabolism, with astrocytes being the main CNS cells responsible for oxidizing fatty acids [27]. There is limited information on the effects of palmitate on the brain, with most studies focusing on hypothalamic neurons since they sense the nutritional and metabolic information of the body. It has been shown that palmitate causes insulin resistance in vivo and in vitro in hypothalamic neurons, but few studies have addressed the effect of palmitate on insulin resistance in glial cells, and the mechanisms underlying such an effect remain to be determined. Astrocytes from different areas of the CNS respond differently to palmitate treatment. Astrocyte cells are morphologically complex, and their characteristics depend on the CNS area to which they belong.

In our study, it was demonstrated that in an astrocyte cell line from rats (C6) which received short-term treatment with palmitate (C16:0), there was a decrease in cell viability in these cells, indicating a cytotoxic effect of palmitate. Similar results were found in an in vitro model of the T98G astrocyte line, which after 48 h of treatment with palmitate exhibited drastically reduced cell viability in a dose-dependent manner [19]. Other in vitro experimental evidence also suggests that palmitate treatment for 24 h reduces cell viability in astrocytes [28,29,30]. However, when exposing these cells to other saturated fatty acids, such as lauric acid (C12:0) and stearic acid (C18:0), there are no changes in cell viability [19,29], suggesting a specific effect of palmitate and corroborating our results that stearate does not cause changes in cell viability. On the other hand, astrocytes treated with oleate (C18:1), an unsaturated fatty acid, had increased cell viability, without causing a toxic effect [19].

In murine models subjected to a high-fat diet, it has been demonstrated that there is an increase in GFAP-positive cells in the cortex and hippocampus of these animals, suggesting astrogliosis [31,32]. As our cell model is an astrocytic tumor, these cells are positive for GFAP [33], and immunocytochemistry was used to evaluate changes in the morphology of these cells. Stimuli that increase the intracellular content of cyclic adenosine monophosphate (cAMP) (such as forskolin) trigger cell body retraction in C6 cells [33], and it was demonstrated that treatments with 200 µM and 400 µM palmitate increased intracellular cyclic AMP levels in the hepatocellular carcinoma cell line HepG2 [34]. Our results showed that treatment with 200 µM palmitate changed the morphology of astrocytes, which became more rounded. These results culminate in studies in which palmitate caused changes in astrocytic morphology, making them round and thick [28] and also causing nuclear fragmentation [35]. However, stearate did not cause morphological changes.

In addition to the change in morphology, another important factor to indicate astrocytic reactivity is the increase in pro-inflammatory cytokines. Studies have demonstrated that palmitate treatment in astrocytes increases the gene expression of cytokines such as TNF-∝, IL-6, and IL-1β [18,29,30,36], results also verified in our study in which palmitate increased the gene expression of TNF-∝ and IL-6 but not IL-1β. The increase in the release of pro-inflammatory cytokines also indicates that palmitate induces gliosis in our in vitro model. Taken together, the results of changes in morphology and increased gene expression of pro-inflammatory cytokines suggest and corroborate that palmitate causes reactivity and neuroinflammation in astrocytes.

Mitochondrial dysfunction is a key factor found in obesity [10,37]. In murine models of obesity, a decrease in ATP synthesis, an increase in reactive oxygen species, and mitochondrial swelling in the cortex of these animals were observed [38,39,40]. In our study, palmitate decreased cell viability, suggesting a decrease in the enzymatic activity of mitochondrial enzymes, which could be a prediction that mitochondria may be dysfunctional [41]. Palmitate treatment decreased mitochondrial membrane potential in astrocytes, both by the JC-1 and Mitotracker Deep Red probes. Similar results were observed with very high concentrations of palmitate, 1 mM [28] and 2 mM [35], and with long-term treatment (24 h). Furthermore, González-Giraldo et al. (2018) found using acridine orange dye that palmitate decreased mitochondrial mass in astrocytes [28].

Long-chain fatty acids are well-known uncouplers of oxidative phosphorylation due to their protonophoric action [42]. Non-esterified long-chain fatty acids also belong to compounds that promote the opening of the mitochondrial permeability transition pore (PTP), a large non-selective channel that opens under specific conditions as a result of Ca^2+^ accumulation in the mitochondrial matrix compartment [43], and they also promote the opening of a channel for the direct flux of protons (and other ions) across the inner mitochondrial membrane [44]. The effect of palmitate-stimulated respiration occurs through the involvement of the ATP/ADP antiporter [45]. However, the observed effect of palmitate was noted at low concentrations of fatty acids (10–20 µM), while in our model, we used a higher concentration (20 µM) and a longer treatment duration, which could induce a prolonged opening of the PTP. Prolonged opening of the PTP, as a non-selective mitochondrial pore, has been repeatedly linked to mitochondrial dysfunction, resulting in permanent mitochondrial depolarization.

In our study, we also demonstrated that palmitate decreased, in astrocytes, the enzymatic activity of mitochondrial complex I, the NADH-dehydrogenase, without changes in complex II and complex IV. Similarly, in mice treated with 4 weeks of a high-fat diet, there was a decrease in mitochondrial respiration linked to complex I in the hippocampus [46]. In an in vitro model of macrophages (Thp-1 cells) treated with 500 µM of palmitate for 24 h, there was a decrease in the activity of complex II and IV [47]. Therefore, we may not have evidenced changes in these enzymatic activities because the astrocytes were treated with a lower concentration and for a shorter time.

In vivo models treated with high-fat diets show a decrease in mitochondrial oxygen consumption in the hippocampus of animals [9,46]. In addition, Hansen et al. (2021) observed that primary endothelial cells treated with high concentrations of palmitate and high concentrations of glucose for three days resulted in a decrease in mitochondrial respiration and an increase in reactive oxygen species [48]. However, in our study, astrocytes treated with palmitate did not show changes in the different stages and parameters of mitochondrial respiration, again highlighting that it may have been influenced by the time and concentration of exposure.

Studies have found that high-fat diets alter the homeostasis of mitochondrial fusion and fission in the brain tissues of murine models [49,50,51]. Maneechote et al. (2022) demonstrated that Wistar rats exposed to a high-fat diet had changes in the content of proteins related to mitochondrial dynamics in hippocampal and cortex homogenates, increasing the protein content of Drp1, decreasing the protein content of Mfn2, and exhibiting unchanged Opa1 [49]. In our work, we also found that palmitate decreased Mfn2 gene expression in astrocytes, but Opa1 expression remained invariable. Thus, high-fat diets and high concentrations of palmitate can alter the homeostasis of mitochondrial dynamics (apparently increasing fission and decreasing fusion) in brain cells, triggering mitochondrial dysfunction. Citrate synthase plays a crucial role in cellular metabolism. Its abundance and activity are often correlated with mitochondrial content and energy production capacity. In our study, there was a decrease in citrate synthase gene expression, suggesting a decrease in mitochondrial mass. This finding together with the finding of González-Giraldo et al. (2018) indicates that palmitate decreases mitochondrial fusion and consequently decreases mitochondrial mass in astrocytes [28].

Mitophagy is the removal of dysfunctional mitochondria through autophagy, which in mammals is preceded by mitochondrial fission [52]. In our study, we only evaluated the gene expression of a single protein (beclin) involved in autophagy, and we did not observe changes in transcript levels. Chen et al. (2018) determined that astrocytes treated with 250 µM palmitate for 12 h increased the protein content of microtubule-associated protein 1A/1B-light chain 3 conjugated with phosphatidylethanolamine (LC3B-II), a protein involved in the formation of the autophagosome [36]. In the same study, it was observed by electron microscopy that palmitate increased the number of autophagosomal vesicles in astrocytes [36]. Also, Ortiz-Rodriguez et al. (2019) demonstrated that palmitate increased the protein content of LC3B-II and p62 (a protein also involved in autophagy) in astrocytes [18]. However, palmitate decreased autophagic flux, suggesting the suppression of autophagy and accumulation of autophagosomal vesicles without their complete degradation, culminating in cell death by apoptosis and necrosis [18].

Oxidative stress is defined as a disturbance in the balance between the production of reactive oxygen species (free radicals) and antioxidant defenses. Evidence from the literature shows that palmitate causes an increase in the production of reactive oxygen species in astrocytes [30,35,53]. Despite this, our results showed that short-term treatment with palmitate did not cause an increase in ROS and corroborated another study in which palmitate did not induce oxidative stress in astrocytes [28]. Also, we did not observe an increase in the production of species reactive to thiobarbituric acid, notwithstanding literature results showing an increase in TBARS in astrocytes treated for 24 h with palmitate [19].

Palmitic acid is a saturated fatty acid whose blood concentration is elevated in obese patients. This elevation causes inflammatory responses, in which TLR2 and TLR4 play important roles. Inside the cell, palmitate is converted into phospholipids, diacylglycerol, and ceramides, triggering the activation of various signaling pathways common to LPS-mediated TLR4 activation. Specifically, these pathways affect the activation of various protein kinase C (PKCs), induce Endoplasmic reticulum (ER) stress, and lead to an increase in ROS generation [29,53]. Stearic acid, one of the most common fatty acids in brain phospholipids, originates in the circulation and is sequestered from the blood exclusively by the brain, along with precursor fatty acids. The high concentration of stearic acid in brain gray matter suggests that this fatty acid plays an important role in neural function. Recently, it has been found that several fatty acids can function as natural ligands of peroxisome proliferator-activated receptors (PPARs) to regulate lipid homeostasis. PPARα and PPARγ have modulatory roles in various cellular responses, including immune and inflammatory responses. It has been reported that ligands of PPARα or PPARγ can significantly protect the brain from ischemic or oxidative insult. Stearate can effectively protect cortical neurons against oxidative stress, possibly mediated mainly by the activation of PPARγ and the synthesis of new proteins in cortical neurons. In contrast to palmitate, stearate has a neutral or cholesterol-lowering effect, which differs from the effects of lauric, myristic, and palmitic acids, both in humans and in experimental animals [53].

Non-esterified fatty acids are primarily transported with serum albumin, and albumin–FA interactions enhance the amount of FA transported and cleared from circulation. Studies conducted using primary hepatocyte cell cultures demonstrate that, in the presence of albumin, palmitate uptake by cells is more efficient. In vitro systems reveal that albumin-promoted FA delivery follows the ‘pseudofacilitation model’, in which FAs are delivered as a function of their unbound concentration in the medium at a physiological concentration of albumin (~200 µM). At lower concentrations (<150 µM), delivery will depend on the albumin concentration [20].

The selection of the type of BSA used in cellular lipotoxicity models requires careful consideration, as differences in isolation methods, purity, and contaminants can potentially interfere with the biological effects of lipid preparations. In our study, we utilized fatty acid-free albumin; however, we acknowledge that BSA contaminants, such as hormones, drugs, and toxins, may remain bound to albumin after isolation. To minimize the impact of BSA, we included a control group treated solely with BSA to assess any potential effects on the parameters measured. As illustrated in all figures, BSA alone had no discernible effect on cell viability, morphology, inflammatory markers, or mitochondrial parameters.

Therefore, in conditions of obesity, saturated fatty acids circulating in plasma can cross the BBB and primarily affect astrocytes, causing metabolic imbalance in mitochondrial function and impacting the viability of these cells, which will consequently cause damage to CNS homeostasis. As a result of these events, astrocytes, the cells responsible for nourishing and supporting neurons, will be under stress, which can trigger neuronal dysfunction and contribute to the development of neurodegenerative diseases.

## 5. Conclusions

We can conclude that high concentrations of palmitate caused mitochondrial dysfunction in an in vitro model of astrocytes by decreasing the mitochondrial membrane potential, decreasing the enzymatic activity of complex I, decreasing the expression of proteins involved in mitochondrial fusion, and decreasing mitochondrial mass, generating an imbalance in mitochondrial metabolism that may reflect the functional state of the respiratory chain. Furthermore, astrocytes treated with palmitate exhibited an increased gene expression of pro-inflammatory mediators and changes in morphology, suggesting that palmitate causes astrogliosis. As a result of these events, mitochondrial dysfunction culminates in a decrease in cell viability. However, stearate did not cause changes in any of the parameters evaluated, indicating the individual effect of palmitate.

Changes in mitochondrial metabolism in astrocytes can generate a metabolic imbalance in the central nervous system, leading to cellular dysfunction in conditions of obesity and thus favoring the development of neurodegenerative diseases.

## Figures and Tables

**Table 1:** Gene bank numbers of primers.

Gene	Gene ID
Gapdh	14433 transcript NM_001411840.1
Il1b	16176 transcript NM_008361.4
Il6	16193 transcript NM_031168.2
Tnf	21926 transcript NM_013693.3
Mfn2	170731 transcript NM_133201.3
CS	12974 transcript NM_026444.4
Acaca	107476 transcript NM_133360.3
Opa1	74143 transcript NM_133752.4
Becn1	56208 transcript NM_019584.4

## Data Availability

The raw data supporting the conclusions of this article will be made available by the authors on written request. Please submit a written request to the authors for the raw and/or processed data files. The data are not publicly available due to privacy.

## Abbreviations

ANOVA	Analysis of variance
ATP	Adenosine triphosphate
BBB	Blood–brain barrier
BMI	Body mass index
BSA	Bovine serum albumin
CNS	Central nervous system
DAPI	4′,6-diamidino-2-phenylindole
DCF	Dichlorofluorescein
DCFH	Dichlorodihydrofluorescein
DCFH-DA	2,7-dichlorodihydrofluorescein diacetate
DCIP	2,6-dicloindophenol
DMEM	Dulbecco’s modified Eagle’s medium
DMSO	Dimethylsulfoxide
FBS	Fetal bovine serum
FFA	Free fatty acids
GAPDH	Glyceraldehyde-3-phosphate dehydrogenase
GFAP	Glial fibrillary acid protein
GLUT1	Glucose transporter 1
HFD	High-fat diet
IL-1β	Interleukin 1 β
IL-6	Interleukin 6
LC3B-II	Microtubule-associated protein 1A/1B-light chain 3 conjugated with phosphatidylethanolamine
Mfn2	Mitofusin-2
MTT	3-(4,5-dimethylthiazol-2-yl)-2,5-diphenyltetrazolium
NADH	Nicotinamide adenine dinucleotide
PA	Palmitate
PBS	Phosphate-buffered saline
ROX	Extramitochondrial respiration
SEM	Standard error of the mean
Ste	Stearate
TBARS	Thiobarbinturic-acid-reactive substances
TNF-α	Tumor necrosis factor-α
UCP-2	Uncoupler protein-2
ROS	Reactive oxygen species
